# The tiny-leaved orchid *Disperis neilgherrensis* primarily obtains carbon from decaying litter via saprotrophic *Ceratobasidium*

**DOI:** 10.1007/s00572-025-01183-x

**Published:** 2025-02-13

**Authors:** Kenji Suetsugu, Ryuta Yagi, Hidehito Okada, Jun Matsubayashi

**Affiliations:** 1 Department of Biology, Graduate School of Science, Kobe University, 1-1 Rokkodai, Nada-Ku, Kobe, Hyogo 657-8501 Japan; 2 Institute for Advanced Research, Kobe University, 1-1 Rokkodai, Nada-Ku, Kobe, Hyogo 657-8501 Japan; 3 Faculty of Marine Science and Technology, Fukui Prefectural University, 1-1 Gakuen-Cho, Obama, Fukui 917-0003 Japan

**Keywords:** Carbon dynamics, Mixotrophy, Mycorrhizas, Partial mycoheterotrophy, Rhizoctonias, Saprotrophic fungi

## Abstract

While most green orchids establish associations with non-ectomycorrhizal rhizoctonias belonging to Ceratobasidiaceae, Tulasnellaceae, and Serendipitaceae, fully mycoheterotrophic orchids—excluding albino mutants—primarily depend on either ectomycorrhizal fungi or saprotrophic non-rhizoctonia fungi. This suggests that non-ectomycorrhizal rhizoctonias may be unable to meet the carbon demands of adult orchids that exhibit a high degree of mycoheterotrophy. To understand the physiological ecology of *Disperis neilgherrensis*, an orchid species with reduced leaves growing in decaying litter from non-ectomycorrhizal trees, we employed molecular and stable isotope analyses to identify its mycorrhizal partners and ultimate nutritional sources at two populations on Ishigaki Island, Japan. Molecular barcoding techniques revealed that *D. neilgherrensis* forms exclusive associations with non-ectomycorrhizal Ceratobasidiaceae fungi. The *Disperis* specimens exhibited δ^13^C and δ^15^N isotopic values similar to those found in fully mycoheterotrophic orchids that exploit litter-decaying fungi. Furthermore, the pelotons of *D. neilgherrensis* showed significantly elevated δ^13^C values similar to saprotrophic non-rhizoctonia fungi. Our findings indicate that *D. neilgherrensis* primarily obtains its carbon from decaying litter through a specialized relationship with non-ECM Ceratobasidiaceae. Given that saprotrophic Ceratobasidiaceae facilitate nearly fully mycoheterotrophic growth in *D. neilgherrensis*, at least under warm and humid conditions, it is plausible that other (nearly) fully mycoheterotrophic tropical orchids also meet their carbon requirements through associations with saprotrophic rhizoctonias.

**Supplementary Information:**

The online version contains supplementary material available at 10.1007/s00572-025-01183-x.

## Introduction

The majority of plants engage in a mutualistic exchange, sharing photosynthates with fungi to secure essential inorganic, and sometimes organic, nutrients such as nitrogen and phosphate through mycorrhizal symbiosis (Smith and Read 2008; Heijden et al. 2015). Orchids, however, diverge from this common framework. In their initial life stages, orchid species exhibit mycoheterotrophy due to the absence of endosperm in their seeds and limited carbon reserves in their embryos, a phenomenon referred to as initial mycoheterotrophy (Hynson et al. 2013).

It is also well-known that some green orchids, including *Cephalanthera* and *Epipactis*, exhibit a nutritional strategy combining photosynthates and fungal organic carbon during their mature phases, possibly to compensate for the shortage of photosynthesis-derived carbon under their dark forest understory habitats (Gebauer and Meyer 2003; Bidartondo et al. 2004; Julou et al. 2005). The phenomenon of partial mycoheterotrophy in these green orchids is principally evidenced by their reflection of the isotopic composition of associated fungal symbionts, conspicuously displaying enhanced enrichment in the isotopes ^13^C and ^15^N when contrasted with carbon fixed through photosynthetic processes and inorganic nitrogen procured from soil substrates (Gebauer and Meyer 2003; Bidartondo et al. 2004; Julou et al. 2005).

The prevailing hypothesis suggests that the transition from autotrophy to full mycoheterotrophy occurs progressively through intermediate phases (Selosse and Roy 2009; Jacquemyn and Merckx 2019). This notion is supported by the close phylogenetic relation between partially mycoheterotrophic orchids and their fully mycoheterotrophic counterparts (Selosse and Roy 2009; Motomura et al. 2010; Jacquemyn and Merckx 2019). Moreover, it is reasonable to assume that partially mycoheterotrophic plants evolved from ancestors that initially exhibited mycoheterotrophy during germination (Selosse and Roy 2009; Hynson et al. 2013; Jacquemyn and Merckx 2019). These insights propose a two-step evolutionary pathway to complete mycoheterotrophy, involving a shift from initial to partial mycoheterotrophy, followed by a subsequent progression from partial to full mycoheterotrophy (Selosse and Roy 2009; Hynson et al. 2013; Jacquemyn and Merckx 2019).

Thus, while green plants closely related to fully mycoheterotrophic species offer promising candidates for studying partial mycoheterotrophy, this phenomenon may also be present in plant groups (e.g., *Disperis*) that exhibit initial mycoheterotrophy but lack fully mycoheterotrophic species (Gebauer et al. 2016; Suetsugu et al. 2020b). Here, we focused on *Disperis neilgherrensis*, which occupies dark forest understory habitats and has reduced leaves. These characteristics have led to a suspicion of partial reliance on fungal-derived carbon (Hashizume 2023), although the hypothesis has not yet been empirically tested.

The genus *Disperis* includes a group of terrestrial orchids in the subfamily Orchidoideae, with a significant presence across Africa, Madagascar, and the neighboring Indian Ocean islands, encompassing around 80 species (Kurzweil 2005; Kurzweil and Manning 2005; Pailler et al. 2019). Its range also spreads to tropical Asia, extending from India to Thailand, the Philippines, and New Guinea (Kurzweil 2005; Kurzweil and Manning 2005; Pailler et al. 2019). The taxonomic revision of Asian species resulted in the recognition of a single widespread *Disperis* species, *D. neilgherrensis*, in Asia, leading to the synonymization of as many as 10 species (Kurzweil 2005).

Despite being initially classified in the subtribe Coryciinae, recent molecular evidence indicates that *Disperis* is more closely related to *Brownleea* than to the subtribe Coryciinae (Waterman et al. 2009). *Disperis* was subsequently repositioned under the subtribe Brownleeinae (Chase et al. 2015). Remarkably, the genus harbors probable nearly fully mycoheterotrophic species such as the leafless African species *D. breviloba*, which retains chlorophyllous stems (Kurzweil and Manning 2005), even though both *Brownleea* and *Disperis* lack fully mycoheterotrophic plants (Merckx et al. 2013). While these features serve as indicative markers for the presence of partial mycoheterotrophy within the genus, the isotopic characteristics, which could further elucidate this association, remain unexamined across any species of the subtribe Brownleeinae.

We also note that the evolutionary trajectory of mycoheterotrophy is frequently associated with shifts in mycorrhizal fungal partners (Bidartondo et al. 2004; Ogura-Tsujita et al. 2012; Yagame et al. 2016). Most green-leaved orchids establish associations with non-ectomycorrhizal rhizoctonias, a polyphyletic group encompassing the families Ceratobasidiaceae, Sebacinales, and Tulasnellaceae. In contrast, not only fully mycoheterotrophic orchids but also those partially mycoheterotrophic with a pronounced degree of heterotrophy are predominantly associated with either ectomycorrhizal or non-rhizoctonia saprotrophic fungi (Selosse and Roy 2009; Motomura et al. 2010; Hynson et al. 2013; Jacquemyn and Merckx 2019; Suetsugu and Matsubayashi 2021a; Suetsugu et al. 2021b, 2022). Given the absence of fully mycoheterotrophic adult orchids in association with non-ECM rhizoctonias—with the exception of albino mutants—a transformation in fungal associations is frequently regarded as an essential precondition for attaining full mycoheterotrophy.

In contrast, the mycorrhizal communities associated with all the 14 *Disperis* taxa previously investigated in South Africa have been dominated by the non-ECM clade of Ceratobasidiaceae (Waterman et al. 2011), although ECM-forming capability has evolved twice within Ceratobasidiaceae (Veldre et al. 2013). However, it should be noted that some *Disperis* species, including *D. neilgherrensis*, are predominantly situated in dark forest understories, whereas these South African species are frequently found in more open environments, such as grasslands or sclerophyllous scrub (Kurzweil and Manning 2005). Given that mycoheterotrophy is considered an adaptation for survival in low-light conditions (Bidartondo et al. 2004; Selosse and Roy 2009; Jacquemyn and Merckx 2019), the fungal associations and degree of mycoheterotrophy might vary between *D. neilgherrensis* and its South African counterparts. Actually, *Epipactis* species in dark forest understories typically associate with ectomycorrhizal fungi and exhibit partial mycoheterotrophy, whereas those in grasslands maintain associations with non-ECM rhizoctonias and autotrophy (Bidartondo et al. 2004; Lallemand et al. 2018; Jacquemyn et al. 2021).

Therefore, considering its habitat in the dark forest understory and reduced leaves, *D. neilgherrensis* might exhibit increased fungal dependency compared to the South African *Disperis* taxa. This could lead to a shift towards associations with ECM-forming Ceratobasidiaceae or other ECM partners. Alternatively, if *D. neilgherrensis* aligns with non-ECM Ceratobasidiaceae, it may represent a rare example of rhizoctonia-associated orchids with a high degree of mycoheterotrophy (Suetsugu et al. 2025). Given that ECM trees are often not present in the natural habitat of *D. neilgherrensis*, we lean towards the latter possibility. To test the hypothesis, our investigation focused on (i) determining whether *D. neilgherrensis* relies on non-ECM rhizoctonia or ECM fungi, based on high-throughput DNA sequencing, and (ii) establishing whether *D. neilgherrensis* exhibits a high degree of mycoheterotrophy, based on ^13^C and ^15^N isotope analysis.

## Materials and methods

### Study species and sampling localities

**Fig. 1:**
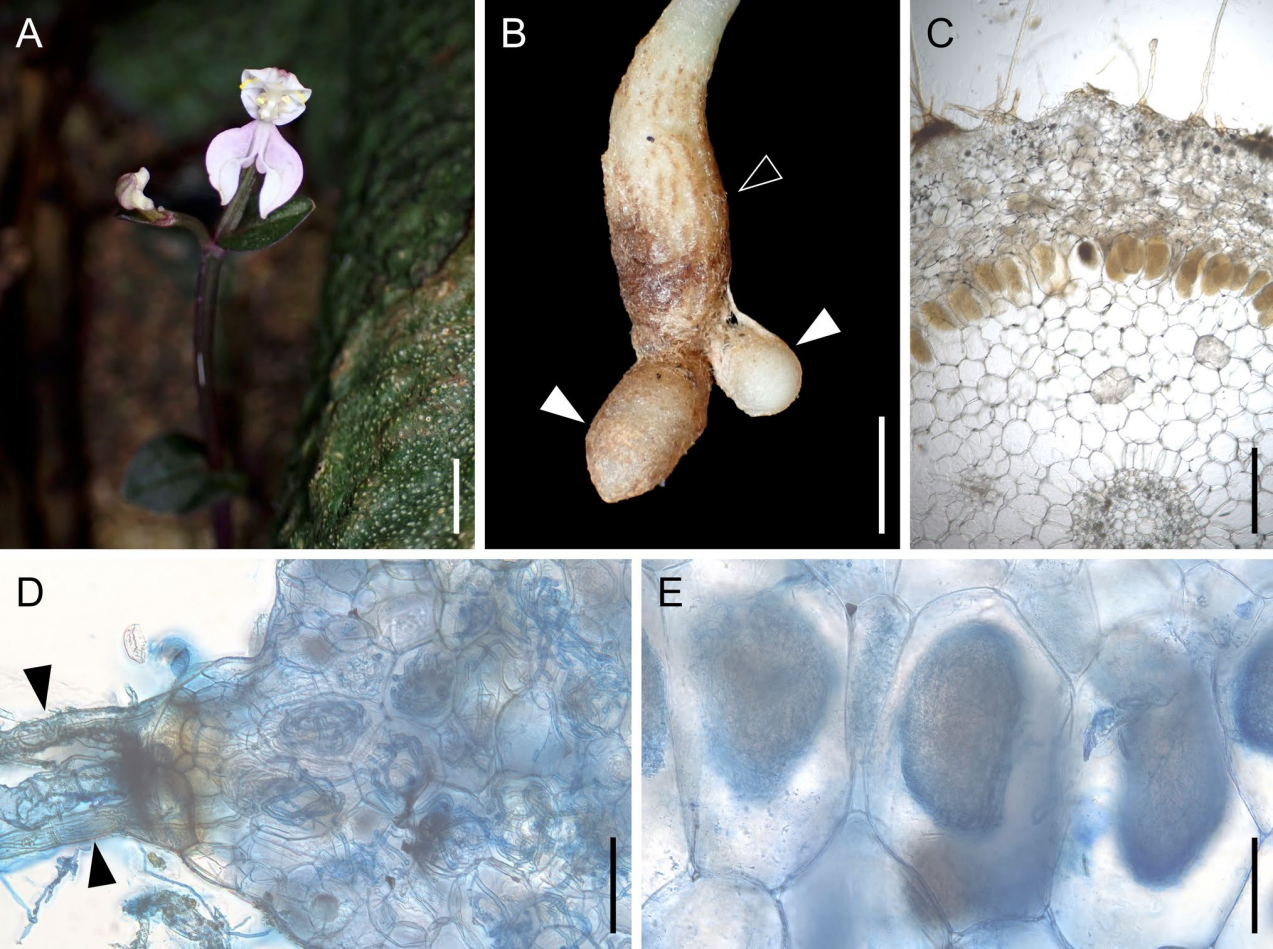
*Disperis neilgherrensis* and its mycorrhizal interaction. (**A**) Flowering plant. (**B**) Underground system bearing rhizome (black arrow) and tubers (white arrows). (**C**) Cross section of rhizome. (**D**) Cross section of rhizome with undegenerated fungal coils stained with trypan blue. Colonization of mycorrhizal fungi was initiated from rhizome hairs (black arrows). (**E**) Close-up of rhizome cells with degenerated fungal coils stained with trypan blue. Scale bars: 5 mm (**A**–**B**), 500 μm (**C**), 150 μm (**D**), and 100 μm (**E**)

*Disperis neilgherrensis* is a diminutive terrestrial orchid found in the shaded understory of (sub)tropical forests. The subterranean structure of *D. neilgherrensis* comprises a rhizome and tubers. Near the plant base, a tuber enclosed in a brownish covering develops, devoid of any evidence of mycorrhizal fungal presence. In contrast, the rhizome, positioned between the tuber and the flowering stem, displays mycorrhizal colonization within its outer and middle cortical cells (Fig. 1).

In August 2023, a field study was conducted in two populations (Fukai and Ishigaki) on Ishigaki Island, Okinawa Prefecture, Japan. Both study sites were subtropical forests predominantly characterized by trees associated with arbuscular mycorrhizal fungi, and no ECM trees were observed. At the Fukai population, the dominant tree species included *Diospyros maritima*, *Garcinia subelliptica*, *Ficus macrocarpa*, *Machilus thunbergii*, *Arenga engleri*, *Psychotria asiatica*, and *Psychotria manillensis.* The Ishigaki population was covered by *Ficus superba*, *Ficus variegata*, *Melia azedarach*, *Tetradium glabrifolium*, *Heptapleurum heptaphyllum*, *Arenga engleri*, and *Morus australis*. All *D. neilgherrensis* plants were found growing in decomposing leaf litter from these non-ECM trees. During the investigation year, there were approximately 50 flowering individuals of *D. neilgherrensis* at both populations. The two populations are located approximately 10 km apart. Apart from *D. neilgherrensis,* a few plant species, including *Dioscorea pseudojaponica* and *Codonacanthus pauciflorus*, were widely distributed across the forest floor within the study sites.

We established four 1-m by 1-m quadrats containing a *D. neilgherrensis* flowering individual at the Fukai population and three at the Ishigaki population. Consequently, underground samples from seven *D. neilgherrensis* individuals were collected for the molecular barcoding of mycobionts. Concurrently, the aboveground parts of these individuals were harvested for ^13^C and ^15^N isotope analysis. In addition, we gathered leaves from at least three co-occurring autotrophic reference species within each quadrat for isotope analysis. To minimize the influence of environmental variables, such as atmospheric CO_2_ isotope composition, microscale light conditions, and soil type (Gebauer and Schulze 1991), we collected the leaves from reference plants growing at the same height as the focal *D. neilgherrensis* individuals.

### Molecular identification of mycobionts

Rhizomes were excised and examined under a light microscope to confirm mycorrhizal colonization. For molecular analysis, mycorrhizal fragments containing fungal pelotons (approximately 3 mm in length) were collected from each sample. To remove potential contaminants, the mycorrhizal samples underwent surface sterilization. DNA extraction from the sterilized samples followed the cetyltrimethylammonium bromide method (Doyle and Doyle 1990).

The ITS region sequences of mycorrhizal fungi were amplified using the primer set ITS86F/ITS4, which is appropriate for studying orchid root fungal communities, despite some primer bias against *Tulasnella* (Waud et al. 2014). These primers were fused with 3–6-mer Ns and Illumina forward/reverse sequencing primers as detailed in Suetsugu et al. (2021a). PCR was conducted using the Q5 High-Fidelity DNA Polymerase kit with the following conditions: initial denaturation at 98 °C for 40 s, followed by 35 cycles of 98 °C for 5 s, 58 °C for 10 s, and 72 °C for 20 s, concluding with a final extension at 72 °C for 10 min. A supplemental PCR was carried out to incorporate the Illumina P5/P7 adapter sequences and sample-specific indices (Syed et al. 2009; Suetsugu and Matsubayashi 2021b). Conditions for the supplemental PCR were as follows: initial denaturation at 98 °C for 40 s, followed by 12 cycles of 98 °C for 5 s, 65 °C for 10 s, and 72 °C for 20 s, and a final extension at 72 °C for 10 min. The pooled library was sequenced using the Illumina MiSeq sequencer with the MiSeq Reagent Micro Kit v2 (300 cycles). The sequence data have been deposited in the NCBI Sequence Read Archive under accession no. PRJNA1164430.

After sequencing, bioinformatic analysis was conducted using Claident v0.9.2020.12.06 (Tanabe and Toju 2013), as outlined by Suetsugu & Okada (2021). In summary, this process involved the removal of primer regions, elimination of low-quality reads, and denoising of erroneous sequences using DADA2 (Callahan et al. 2016) implemented in Claident. Subsequently, the sequences possibly arising from PCR chimera formation and index-hopping were removed using the clremovechimev and clremovecontam commands in Claident (Esling et al. 2015; Nilsson et al. 2019). Remaining sequencing reads were clustered into operational taxonomic units (OTUs) at a 97% similarity threshold using VSEARCH v2.8.0 (Rognes et al. 2016) implemented in the clclassseqv command. The most abundant sequence within each OTU cluster was selected as the representative sequence. The taxonomic assignment of the OTUs was conducted using the query-centric auto-*k*-nearest-neighbor (QCauto) and lowest common ancestor (LCA) algorithms (Huson et al. 2007), with the "overall_genus" reference database in Claident. For further analysis, only OTUs identified as potentially orchid mycorrhizal fungi (Dearnaley et al. 2012; Wang et al. 2021) were retained.

Since all *D. neilgherrensis* specimens were predominantly colonized by Ceratobasidiaceae OTUs, we performed a phylogenetic analysis to investigate this relationship further. Specifically, the OTUs identified as mycobionts of *D. neilgherrensis* were subjected to a BLAST query against the International Nucleotide Sequence Database Collaboration (INSDC) for comparative purposes (Altschul et al. 1997). Subsequently, we retrieved several sequences that were closely related phylogenetically, along with other representative sequences from the family Ceratobasidiaceae. These sequences were aligned using ClustalW, and a maximum-likelihood phylogenetic tree was constructed using MEGA X (Kumar et al. 2018) with a GTR + I + G model and 1,000 bootstrap replicates (ln *L* = − 1770.01).

### δ^13^C and δ^15^N analysis

Mycoheterotrophic orchids typically manifest elevated relative ^13^C and ^15^N abundance in comparison to co-occurring autotrophic plants, reflecting the augmented δ^13^C and δ^15^N values inherent to their fungal hosts. Therefore, to infer the nutritional mode of *D. neilgherrensis*, we conducted the δ^13^C and δ^15^N analysis of *D. neilgherrensis* and its co-occurring autotrophic plants.

The quantification of partial mycoheterotrophy can be further refined through the comparative isotope analysis of fruiting bodies of mycobionts (Trudell et al. 2003; Julou et al. 2005). Nevertheless, challenges arise when dealing with rhizoctonia, as they do not yield discernible fruiting bodies. To overcome this challenge, we also obtained intracellular hyphae extracted from three *D. neilgherrensis* rhizomes in the Ishigaki population (Gomes et al. 2023; Zahn et al. 2023). Subsequently, the collected leaves and fungal pelotons underwent desiccation at 60 °C and were finely ground using an agate mortar.

The carbon and nitrogen stable isotope ratios in these samples were measured using a continuous-flow isotope-ratio mass spectrometer (Delta V Advantage; Thermo Fisher Scientific, Waltham, Massachusetts, USA) coupled to an elemental analyzer (Flash EA 2000; Thermo Fisher Scientific, Waltham, Massachusetts, USA), following the protocol outlined by Suetsugu and Matsubayashi (2021b).

The relative isotope abundances were calculated as follows:

$$\mathrm\delta^{13}\mathrm C\;\mathrm{or}\;\mathrm\delta^{15}\mathrm N\left[\permille\right]=\left({R}_\text{sample}/{R}_\text{standard}-1\right)\times1,000,$$

where *R*_sample_ represents the ^13^C/^12^C or ^15^N/^14^N ratio in each sample, and *R*_standard_ represents the ^13^C/^12^C or ^15^N/^14^N ratios of Vienna PeeDee belemnite or atmospheric N_2_, respectively. The C and N isotope ratios were calibrated using the following laboratory standards: CERKU-01 (DL-Alanine, δ^13^C = − 25.36‰, δ^15^N = − 2.89‰), CERKU-02 (L-Alanine, δ^13^C = − 19.04‰, δ^15^N = 22.71‰) and CERKU-03 (glycine, δ^13^C = − 34.92‰, δ^15^N = 2.18‰) (Tayasu et al. 2011). The analytical standard deviations (SDs) obtained from repeated measurements of these standards were less than 0.28‰ for δ^13^C (*n* = 23) and 0.11‰ for δ^15^N (*n* = 37). The total C and N concentrations in the samples were determined by using the measured weights of the samples and the volumes of CO_2_ and N_2_ gases from laboratory standards (Tayasu et al. 2011). Furthermore, we calculated enrichment factors (ε) using the formula ε = δ_Sample_ − δ_REF_, where δ_S_ represents each δ^13^C or δ^15^N value of a *D. neilgherrensis* individual or a *D. neilgherrensis* fungal peloton, and δ_REF_ represents the mean value of all autotrophic reference plants in a specific sampling plot (Preiss and Gebauer 2008).

We divided all samples into three groups: “*D. neilgherrensis*”, “*D. neilgherrensis* fungal pelotons” and “autotrophic reference plants”, and investigated differences in the δ^13^C, δ^15^N, ε^13^C, and ε^15^N values as well as the total C and N concentrations between these groups. To account for potential random effects associated with the sampling plot, we constructed a linear mixed model (LMM) with plot number (Table S1–S2) as a random effect. Each variable was used as the dependent variable, and the group was included as the explanatory variable. In cases where the effects of random intercept were minimal, a simpler linear model without random effects (LM) was employed. Subsequently, post hoc Tukey–Kramer tests were utilized to determine pairwise differences between groups. All statistical analyses were performed under an adjusted significance level of α = 0.008, based on the Bonferroni method for multiple comparisons. The R statistical software (R Core Team 2025) was used for all analyses, with the lme4 package (Bates et al. 2015) employed for the LMM and the multcomp package (Hothorn et al. 2008) for the Tukey–Kramer test.

## Results

### Molecular identification of mycobionts

Community profiling based on the metabarcoding technique revealed that *D. neilgherrensis* consistently associates with a single Ceratobasidiaceae OTU in both populations. All seven specimens were dominated by a single OTU (Ceratobasidiaceae OTU1) assigned to the genus *Ceratobasidium*, accounting for 42,090 reads (99.9% of all reads) in the Fukai population and 27,687 reads (99.7% of all reads) in the Ishigaki population. A second *Ceratobasidium* OTU (Ceratobasidiaceae OTU2) was detected in only one individual from the Fukai population, contributing 23 reads (0.05% of all reads).

**Fig. 2:**
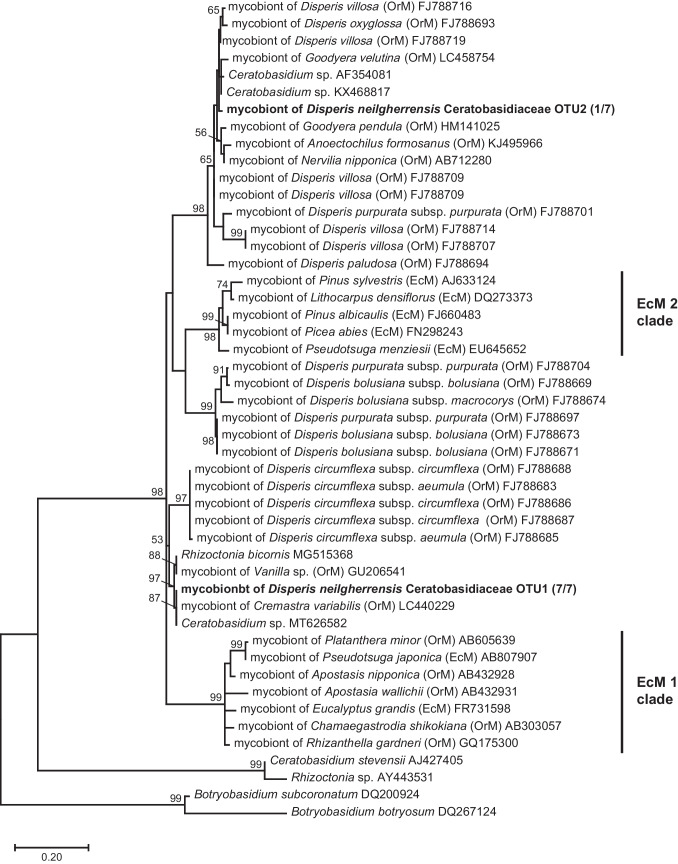
Maximum-likelihood phylogenetic tree of ITS2 rDNA sequences (230 bp) from *Disperis neilgherrensis*-associated fungi and their related taxa. Bold text indicates the sequences obtained in this study. Fractions in parentheses represent the number of individuals in which the respective fungus was detected, relative to the total number of *D. neilgherrensis* individuals analyzed. Other sequences, along with their respective accession numbers, were retrieved from the INSDC database. Node values denote bootstrap support (1,000 replicates) of ≥ 50%

Phylogenetic analysis revealed that the dominant OTU (Ceratobasidiaceae OTU1) formed a strongly supported monophyletic group with mycobionts of some rhizoctonia-associated orchids, but it did not cluster within the same clade as mycobionts previously identified from African *Disperis* species. Conversely, Ceratobasidiaceae OTU2 was found to form a monophyletic clade with mycobionts from several African *Disperis* species studied previously. Although the ectomycorrhiza-forming capability has evolved twice [EcM 1 clade and EcM 2 clade sensu Veldre et al. (2013)] within Ceratobasidiaceae, the OTUs detected in this study were not found to be affiliated with either of these clades (Fig. 2).

### δ^13^C and δ^15^N analysis

**Fig. 3:**
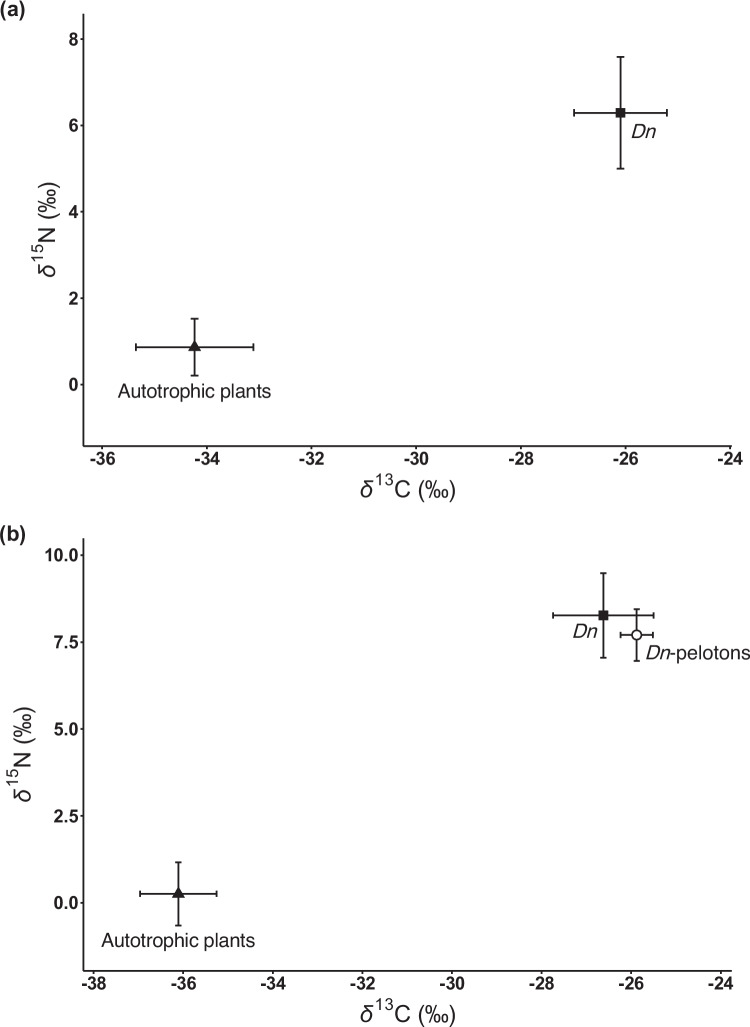
Mean (± SD) δ^13^C and δ^15^N values of *Disperis neilgherrensis* and its neighboring autotrophic plants. (**a**) Fukai population. (**b**) Ishigaki population

The δ^13^C values of *D. neilgherrensis* (− 26.1 ± 0.9‰ in the Fukai population and − 26.6 ± 1.1‰ in the Ishigaki population; mean ± SD) were markedly higher than those observed in autotrophic reference plants (− 34.2 ± 1.1‰ at the Fukai population and − 36.1 ± 0.9‰ at the Ishigaki population; *P* < 0.001; Table S1). Additionally, the δ^15^N values of *D. neilgherrensis* (6.3 ± 1.3‰ at the Fukai population and 8.3 ± 1.2‰ at the Ishigaki population) were significantly elevated compared to those of autotrophic reference plants (0.9 ± 0.7‰ at the Fukai population and 0.3 ± 0.9‰ at the Ishigaki population; P < 0.001; Fig. 3). The ^13^C and ^15^N enrichment factors of *D. neilgherrensis* were calculated to be 8.2 ± 1.4‰ and 5.4 ± 1.4‰ in the Fukai population and 9.5 ± 1.4‰ and 8.0 ± 1.0‰ in the Ishigaki population, respectively. Using the mean value of the ^13^C enrichment factors in fully mycoheterotrophic orchids exploiting litter-decaying, non-rhizoctonia fungi (ε^13^C = 8.7‰) as a fully mycoheterotrophic endpoint (Suetsugu and Matsubayashi 2022), the fungal-derived carbon was estimated to constitute 94.0 ± 15.7% and 108.8 ± 16.0% of the total carbon in the Fukai and Ishigaki populations, respectively. Furthermore, the total nitrogen concentrations in *D. neilgherrensis* leaves (3.6 ± 0.4 mmol/g in the Fukai population and 3.0 ± 0.0 mmol/g in the Ishigaki population) were significantly higher than those found in leaves from autotrophic reference plants (1.7 ± 0.3 mmol/g in the Fukai population and 1.9 ± 0.3 mmol/g in the Ishigaki population; *P* < 0.001).

Notably, the δ^13^C values of fungal pelotons from *D. neilgherrensis* (− 25.9 ± 0.4‰) were also significantly higher than those of autotrophic reference plants (*P* < 0.001; Table S2). Moreover, these δ^13^C values did not show a statistically significant difference from those in *D. neilgherrensis* leaves (*P* = 0.52). As a result, no significant disparities were found in ε^13^C values between the pelotons (10.2 ± 0.6‰) and the leaves of *D. neilgherrensis* (*P* = 0.56). Fungal pelotons extracted from *D. neilgherrensis* were also enriched in ^15^N compared to those from autotrophic reference plants (ε^15^N = 7.5 ± 0.8‰; *P* < 0.001). The total nitrogen concentrations in the pelotons (2.7 ± 0.9 mmol/g) were similar to those in the leaves of *D. neilgherrensis* (*P* = 0.60).

## Discussion

All *D. neilgherrensis* plants examined exhibit a predominant association with a Ceratobasidiaceae OTU distinct from the recognized ECM-forming clades. The ^13^C and ^15^N isotopic signatures indicate that the orchid heavily relies on litter-decaying fungi for carbon nutrition. The rhizoctonia fungi, previously thought to be less capable of supplying carbon to adult orchids (Lallemand et al. 2018; Jacquemyn et al. 2021), are likely effective in meeting the carbon needs of *D. neilgherrensis*, at least under warm and humid subtropical conditions.

The *D. neilgherrensis* specimens were predominantly associated with an OTU affiliated with Ceratobasidiaceae. While read count is not always a precise measure of fungal biomass or colonization level, it can provide a rough indication of fungal quantity (Amend et al. 2010; Wang et al. 2024). The consistent detection of this OTU in all seven individuals, combined with its nearly exclusive representation (> 99.7% of all reads across both populations), strongly supports the specialized nature of the interaction between *D. neilgherrensis* and this OTU. Given its exceedingly low sequencing read count, other fungal OTUs, including Ceratobasidiaceae OTU2 (23 reads detected in a *D. neilgherrensis* specimen), are likely opportunistic fungi with no substantial role in the *D. neilgherrensis* carbon demand.

Since all the *Disperis* taxa investigated are predominantly associated with Ceratobasidiaceae fungi (Waterman et al. 2011), this suggests a pattern of phylogenetic conservatism in their mycorrhizal associations (Shefferson et al. 2019). Phylogenetic analysis revealed that the dominant OTU linked to *D. neilgherrensis* was phylogenetically distinct from the recognized ECM clades (Veldre et al. 2013). Thus, the dominant fungi are more likely to be non-ECM. This assumption is further supported by the fact that the OTU is phylogenetically closely related to the mycobionts of other orchid species associated with non-ECM rhizoctonias.

Despite the leafy status of *D. neilgherrensis*, isotope analysis has shown that this orchid species is considerably enriched in ^13^C relative to autotrophic reference plants. Notably, the ^13^C enrichment factor in *D. neilgherrensis* leaves (8.2 ± 1.4‰ in the Fukai population and 9.5 ± 1.4‰ in the Ishigaki population) aligns closely with that of mycoheterotrophic orchids utilizing litter-decaying, non-rhizoctonia fungi (8.7 ± 1.1‰, *n* = 24), and mycoheterotrophic orchids exploiting ectomycorrhizal fungi (8.2 ± 1.3‰, *n* = 94) (Martos et al. 2009; Ogura-Tsujita et al. 2009; Hynson et al. 2013; Lee et al. 2015; Suetsugu et al. 2020a). This pattern strongly supports the hypothesis that *D. neilgherrensis* primarily obtains its carbon nutrition through fungal symbiosis. Such distinct mycoheterotrophic capabilities could compensate for the reduced production of photosynthetic carbon, associated with diminished leaf size and habitation within intensely shaded forest understories.

Although the ^15^N enrichment in *D. neilgherrensis* leaves (5.4 ± 1.4‰ in the Fukai population and 8.0 ± 1.0‰ in the Ishigaki population) is not as pronounced as the ^13^C enrichment, mycoheterotrophic plants relying on saprotrophic fungi tend to exhibit a lower ^15^N enrichment factor (4.8 ± 1.5‰) compared to those dependent on ECM fungi (Martos et al. 2009; Ogura-Tsujita et al. 2009; Lee et al. 2015; Suetsugu et al. 2020a). Therefore, the relatively low ^15^N enrichment in *D. neilgherrensis* indicates not a low degree of mycoheterotrophy but rather the saprotrophic nature of the associated mycobionts (Mayor et al. 2009). This value sharply contrasts with green orchids (e.g., *Platanthera minor* and *Apostasia nipponica*) that associate with ECM-forming Ceratobasidiaceae (11.4 ± 3.0‰ and 21.6 ± 1.6‰) (Yagame et al. 2012; Suetsugu and Matsubayashi 2021b).

The ^13^C and ^15^N isotopic composition in fungal pelotons provides compelling evidence for the pronounced mycoheterotrophy in *D. neilgherrensis* and the saprotrophic nature of its main mycobiont. Typically, partially mycoheterotrophic orchids have ε^13^C values approximately 3‰ lower than those of pelotons due to dilution by ^13^C-depleted photosynthetic carbon (Gomes et al. 2023; Zahn et al. 2023). Therefore, a similar ^13^C enrichment between *D. neilgherrensis* pelotons and leaves indicates that this orchid primarily gains carbon via mycoheterotrophy. The ^13^C enrichment in *D. neilgherrensis* pelotons (ε^13^C = 10.2 ± 0.6‰) markedly differs from the low ^13^C enrichment (1.4–2.4‰) in pelotons of some rhizoctonia-associated orchids in *Anoectochilus*, *Epipactis*, *Ophrys*, and *Orchis* (Gomes et al. 2023; Zahn et al. 2023). Meanwhile, it is very close to the pelotons extracted from another nearly fully mycoheterotrophic orchid *Stigmatodactylus sikokianus* associated with litter-decomposing rhizoctonia fungi (10.4 ± 0.7‰) (Suetsugu et al. 2021a, 2025). The diversity of ^13^C enrichment patterns possibly reflects various trophic modes, including saprotrophs, facultative plant pathogens, root endophytes, and ectomycorrhizal fungi (Dearnaley et al. 2012; Veldre et al. 2013; Selosse and Martos 2014). Since endophytism typically results in significantly lower ^13^C enrichment (Selosse and Martos 2014), the main mycobionts of *D. neilgherrensis* are likely not endophytic.

The ^13^C enrichment is even higher than that in pelotons from ECM-associated orchids *Epipactis atrorubens* (4.3 ± 1.3‰) and *E. leptochila* (6.8 ± 0.7‰) (Zahn et al. 2023), possibly reflecting that δ^13^C values in ectomycorrhizal fungi are about 3‰ lower than in saprotrophic fungi. The ^15^N enrichment in *D. neilgherrensis* pelotons (ε^15^N = 7.5 ± 0.8‰) is also higher than those composed of non-ECM rhizoctonias (ranging from –1.6 to 4.5‰) (Gomes et al. 2023; Zahn et al. 2023). Nonetheless, given that ECM-forming Ceratobasidiaceae fungi exhibit moderately high ^13^C (6.9 ± 0.6‰) and extraordinarily high ^15^N enrichment (11.1 ± 0.3‰), the contrasting ^13^C and ^15^N enrichment pattern (10.2 ± 0.6‰ and 7.5 ± 0.8‰) in *D. neilgherrensis* suggests its primary mycobiont is a litter-decomposing fungus. This is corroborated by the phylogenetic identity of the fungus and the absence of ECM trees at the study sites.

Intriguingly, not only *D. neilgherrensis* but also other rhizoctonia-associated orchids with a high degree of mycoheterotrophy, including albino mutants, show high ^13^C enrichment levels similar to fully mycoheterotrophic orchids exploiting litter-decaying, non-rhizoctonia fungi (8.7 ± 1.1‰) (Suetsugu et al. 2019, 2021a, c; Suetsugu and Matsubayashi 2022). These observations imply that saprotrophic rhizoctonias effectively foster the development of (nearly) fully mycoheterotrophic orchids, although endophytic rhizoctonias may not proficiently enhance mycoheterotrophic growth during the adult phase (Suetsugu et al. 2021a, 2025). The credibility of this idea is likely strengthened by many orchids becoming completely mycoheterotrophic by exploiting saprotrophic fungi (Martos et al. 2009; Ogura-Tsujita et al. 2009; Lee et al. 2015; Suetsugu et al. 2020a). Notably, these orchids primarily exhibit a tropical distribution, with high humidity and temperatures. The abiotic conditions likely allow saprotrophic fungi to acquire sufficient carbon to support mycoheterotrophic growth (Martos et al. 2009; Selosse et al. 2010; Suetsugu et al. 2025).

Overall, despite the usual link between increased mycoheterotrophy and mycorrhizal shifts (Selosse and Roy 2009; Motomura et al. 2010; Hynson et al. 2013; Jacquemyn and Merckx 2019; Suetsugu and Matsubayashi 2021a; Suetsugu et al. 2022), *D. neilgherrensis* primarily obtains carbon from decaying litter via saprotrophic rhizoctonias at our two sites. Our findings and several recent studies (e.g., Suetsugu et al. 2025) suggest saprotrophic mycobionts, including rhizoctonias, can support (nearly) fully mycoheterotrophic status, especially in favorable hot and humid environments. Thus, the tropical genus *Disperis*, with about 80 species, many with reduced foliage (Kurzweil and Manning 2005), likely includes several partially mycoheterotrophic species highly dependent on rhizoctonia fungi. More broadly, many tropical (almost) fully mycoheterotrophic orchids might derive most of their carbon from rhizoctonias.

## Supplementary Information

Below is the link to the electronic supplementary material.

Supplementary file1 (XLSX 18 KB)

## Data Availability

The sequence data have been deposited in the NCBI Sequence Read Archive (accession no. PRJNA1164430). Additional supporting information is available online in the Supporting Information section at the end of the article.
